# Supplementary material for: Ion mobility collision cross-section atlas for known and unknown metabolite annotation in untargeted metabolomics
Source: Nat Commun. 2020 Aug 28;11:4334. doi: 10.1038/s41467-020-18171-8 (PMC7455731; doi:10.1038/s41467-020-18171-8)
Supplement: Supplementary file 1 — Supplementary Information [file 41467_2020_18171_MOESM1_ESM.pdf]

Supplementary information for

Ion Mobility Collision Cross-Section Atlas for Known and Unknown Metabolite Annotation in Untargeted Metabolomics

Zhiwei Zhou[1,2], Mingdu Luo[1,2], Xi Chen[1,2], Yandong Yin[1], Xin Xiong[1], Ruohong Wang[1,2], and Zheng-Jiang Zhu[1,*]

[1] Interdisciplinary Research Center on Biology and Chemistry, Shanghai Institute of Organic Chemistry, Chinese Academy of Sciences, Shanghai, 200032 P. R. China

[2] University of Chinese Academy of Sciences, Beijing, 100049 P. R. China

Corresponding Author

Correspondence should be addressed to Z.J.Z (jiangzhu@sioc.ac.cn)

List of Supplementary Figures

Supplementary Figure 1: Schematic illustration of the 5-step clean-up and standardization of CCS records.

Supplementary Figure 2: Two examples of CCS outliers in data set 10 in Supplementary Table S1.

Supplementary Figure 3: Examples of unified CCS values with different confidence levels.

Supplementary Figure 4: The coverage of chemical diversity for compounds with experimental CCS values in AllCCS.

Supplementary Figure 5: The MRE values of CCS prediction for different ion adducts.

Supplementary Figure 6: The comparison of predicted CCS values for three compounds obtained from AllCCS and other tools.

Supplementary Figure 7: The candidate reduction of the multi-dimensional match using the predicted CCS values and the experimental MS/MS spectral library.

Supplementary Figure 8: The statistics of average candidates and reduction percentages with the addition of CCS match in different database scales.

Supplementary Figure 9: The percentages of rank improvement for correct candidates with the addition of CCS match to the multi-dimensional match in negative ionization mode.

Supplementary Figure 10: Compound examples for demonstrating the use of AllCCS to improve known metabolite annotations.

Supplementary Figure 11: Metabolite annotation with multi-dimensional match for different biological samples.

Supplementary Figure 12: Multi-dimensional match reduces false candidates for different biological samples.

Supplementary Figure 13: Examples of known metabolite annotations with multi-dimensional match in biological samples.

Supplementary Figure 14: Candidate reduction for features with different abundances.

Supplementary Figure 15: The known unknown and unknown unknown metabolites in extended database.

Supplementary Figure 16: The confirmation of identification of ExtDB016054 using synthesized chemical standard.

Supplementary Figure 17: The pathway and structure enrichment analyses.

Supplementary Figure 18: The IM separation for 4 monosaccharide phosphate isomers.

Supplementary Figure 19: Schematic illustration of molecular descriptor (MD) selection.

Supplementary Figure 20: The comparison of different molecular descriptor selection approaches.

Supplementary Figure 21: Schematic illustration of representative structure similarity (RSS) calculation.

Supplementary Figure 22: The optimization of score weight and tolerance of CCS match in multi-dimensional

match.

List of Supplementary Tables

Supplementary Table 1: The collection and standardization of experimental CCS records from publications.

Supplementary Table 2: The definition of confidence levels for CCS values in AllCCS.

Supplementary Table 3: The statistics of ion adducts in the unified CCS database.

Supplementary Table 4: The statistics of super classes of compounds in the unified CCS database.

Supplementary Table 5: Performance evaluation using the external validation sets 1 and 2.

Supplementary Table 6: The comparison of different CCS calculation tools.

Supplementary Table 7: The training set comparison for machine-learning based CCS prediction tools.

Supplementary Table 8: The possible ion adducts in the data cleaning of experimental CCS values.

Supplementary Table 9: The selected molecular descriptors in positive and negative modes.

Supplementary Table 10: The comparison of different molecular descriptor selection approaches.

Supplementary Table 11: Performance evaluation of the prediction model.

Supplementary Table 12: The comparison between support vector regression and multiple linear regression.

Supplementary Table 13: The information of compounds in predicted AllCCS database.

Supplementary Table 14: The parameters for the in-silico MS/MS tools.

Supplementary Table 15: The parameters for LC-IM-MS/MS data processing.

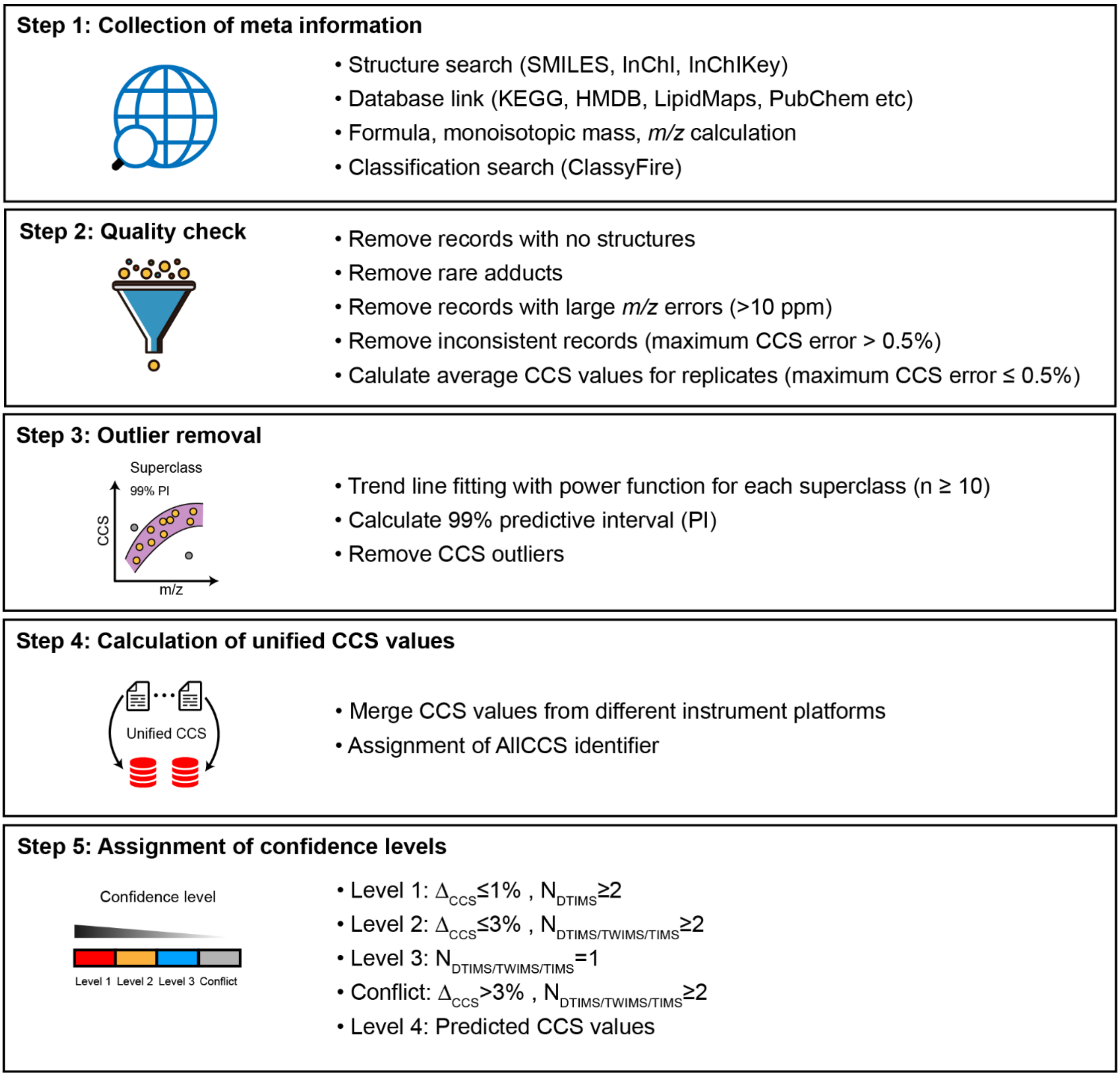

Supplementary Figure 1.

Schematic illustration of the 5-step clean-up and standardization of CCS records.

(1) Collection of meta information; (2) quality check; (3) outlier removal; (4) calculation of unified CCS values; (5) assignment of confidence levels.

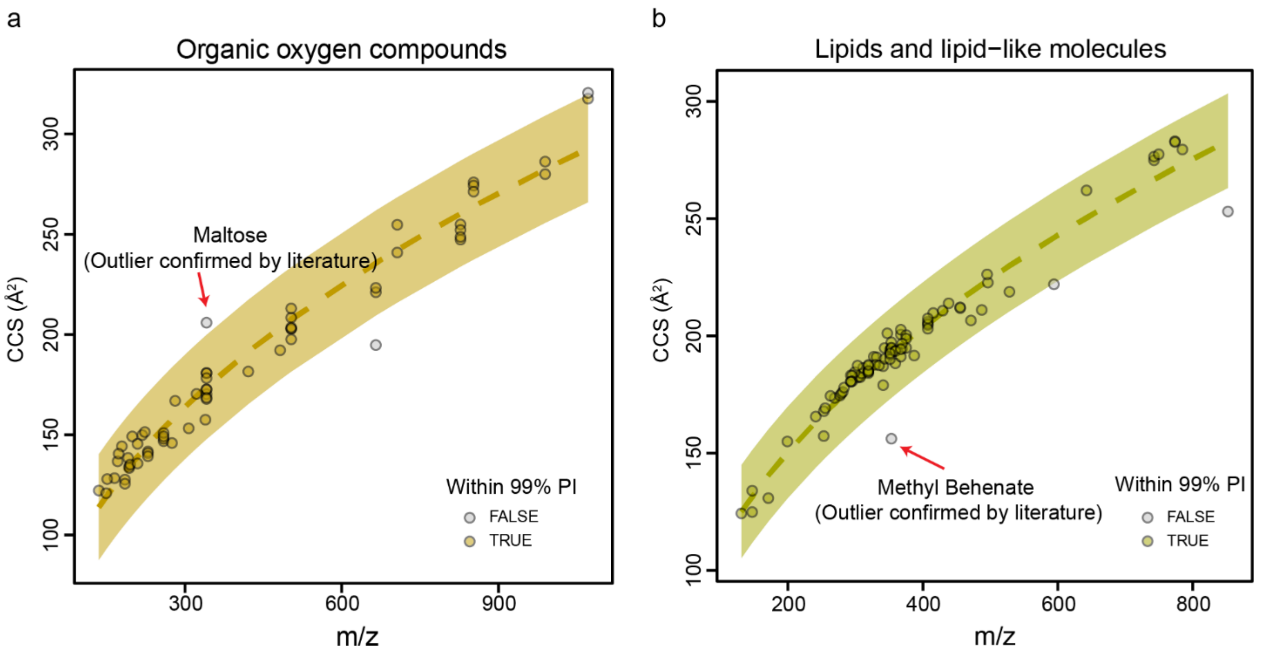

Supplementary Figure 2.

Two examples of CCS outliers in data set 10 in Supplementary Table S1.

(**a**) Maltose was identified as a CCS outlier using the trend line for organic oxygen compounds; (**b**) methyl behenate was identified as a CCS outlier using the trend line for lipids and lipid-like molecules. The error bands represent 99% predictive interval.

a

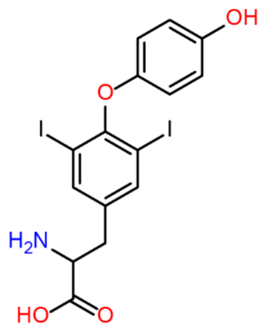

Compound information

AllCCS ID: AllCCS00000021
Names: 3,5-Diiodothyronine
Formula: C15H13I2NO4
Exact mass: 524.8934
InChIKey: ZHSOTLOTTDYIIK-UHFFFAOYSA-N

Experimental CCS records

No.	Adduct	m/z	CCS (Å^2)	Instrument
1	$[M+H]^+$	525.9007	198.7	DTIMS
2	$[M+H]^+$	525.9007	198.3	DTIMS

Unified CCS

No.	Item	Value
1	Adduct	$[M+H]^+$
2	m/z	525.9007
3	CCS (Å^2)	198.5
4	Maxmium error (%)	0.2
5	Reported Labs	2
6	Instrument	DTIMS*2
7	Confidence level	1

b

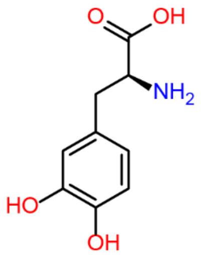

Compound information

AllCCS ID: AllCCS00000007
Names: Levodopa
Formula: C9H11NO4
Exact mass: 197.0688
InChIKey: WTDRDQBEARUVNC-LURJTMIESA-N

Experimental CCS records

No.	Adduct	m/z	CCS (Å^2)	Instrument
1	$[M+H]^+$	198.0761	148.8	DTIMS
2	$[M+H]^+$	198.0761	145.4	TWIMS

Unified CCS

No.	Item	Value
1	Adduct	$[M+H]^+$
2	m/z	198.0761
3	CCS (Å^2)	147.1
4	Maxmium error (%)	2.3
5	Reported Labs	2
6	Instrument	DTIMS*1, TWIMS*1
7	Confidence level	2

c

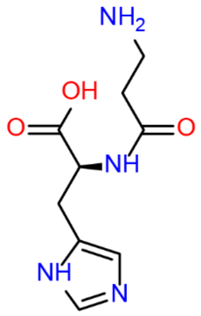

Compound information

AllCCS ID: AllCCS00000003
Names: Carnosine
Formula: C9H14N4O3
Exact mass: 226.1066
InChIKey: CQOVPNPJLQNMDC-ZETCQYMHSA-N

Experimental CCS records

No.	Adduct	m/z	CCS (Å^2)	Instrument
1	$[M-H]^-$	225.0993	149.7	DTIMS
2	$[M-H]^-$	225.0993	154.9	DTIMS
3	$[M-H]^-$	225.0993	152.5	DTIMS
4	$[M-H]^-$	225.0993	152.1	DTIMS

Unified CCS

No.	Item	Value
1	Adduct	$[M-H]^-$
2	m/z	225.0993
3	CCS (Å^2)	152.3
4	Maxmium error (%)	3.5
5	Reported Labs	4
6	Instrument	DTIMS*4
7	Confidence level	Conflict

Supplementary Figure 3.

Examples of unified CCS values with different confidence levels.

(**a**) Level 1: 3,5-Diiodothyronine as $[M+H]^+$; (**b**) level 2: Levodopa as $[M+H]^+$; (**c**) conflict: Carnosine as $[M-H]^-$. The details of CCS records are accessible in AllCCS webserver.

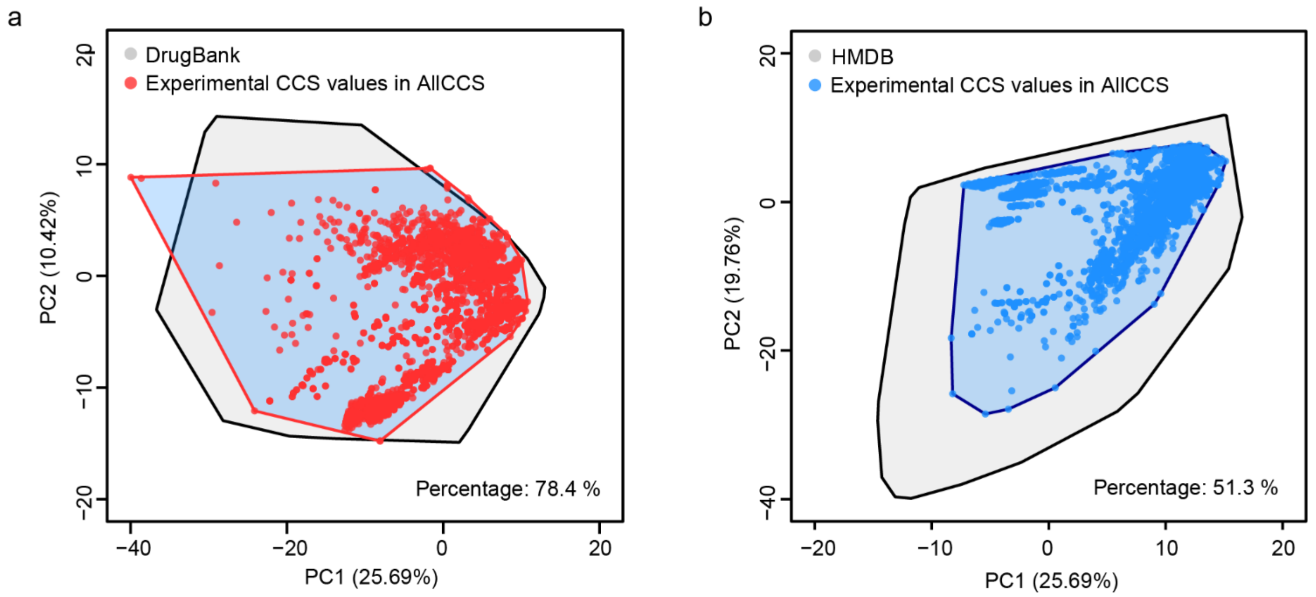

Supplementary Figure 4.

The coverage of chemical diversity for compounds with experimental CCS values in AllCCS.

The chemical diversity of compounds with experimental CCS values compared to all compounds in DrugBank (**a**) and HMDB (**b**). Only small molecules were considered (exact mass ≤ 1200 Da; polymers excluded). Molecular descriptors for each compound were used for principal component analysis (PCA).

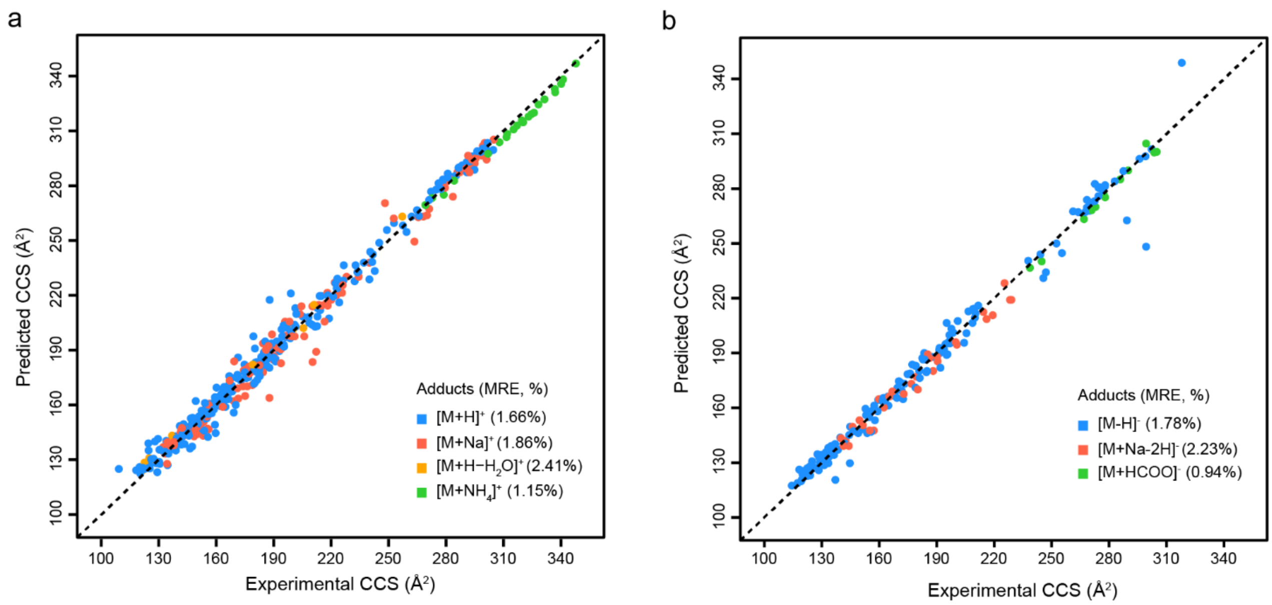

Supplementary Figure 5.

The MRE values of CCS prediction for different ion adducts.
Correlations between the predicted and experimental CCS values in positive (**a**) and negative ionization modes (**b**), respectively. The data was from the external validation set 1 (metabolites & lipids).

a

Metabolite & lipids

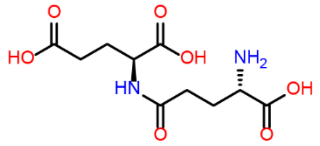

AllCCS ID: AllCCS00000070
Names: gamma-Glutamylglutamate
Formula: C10H16N2O7
Exact mass: 276.0958
Adduct: $[M+H]^+$
m/z: 277.1031
CCS: 162.9 Å^2
InChIKey: OWQDWQKWSLFFFR-WDSKDSINSA-N
Super class: Organic acids and derivatives

No.	Predicted CCS (Å^2)	Rela. error (%)
AllCCS	159.8	1.90
MetCCS	159.4	2.15
DeepCCS	158.6	2.64
ISiCLE	169.8	4.24

b

Metabolite & lipids

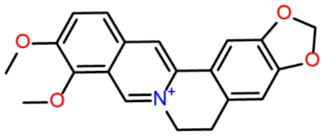

AllCCS ID: AllCCS00001369
Names: Berberine
Formula: C20H18NO4
Exact mass: 336.1236
Adduct: $[M+H]^+$
m/z: 337.1309
CCS: 175.9 Å^2
InChIKey: YBHILYKTIRIUTE-UHFFFAOYSA-N
Super class: Alkaloids and derivatives

No.	Predicted CCS (Å^2)	Rela. error (%)
AllCCS	179.4	1.99
MetCCS	166.0	5.63
DeepCCS	182.5	3.75
ISiCLE	195.2	10.97

c

Natural products & drugs

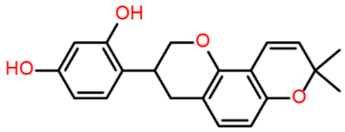

AllCCS ID: AllCCS00028281
Names: (R)-Glabridin
Formula: C20H20O4
Exact mass: 324.1362
Adduct: $[M-H]^-$
m/z: 323.1289
CCS: 184.5 Å^2
InChIKey: LBQIJVLKGVZRIW-ZDUSSCGKSA-N
Super class: Phenylpropanoids and polyketides

No.	Predicted CCS (Å^2)	Rela. error (%)
AllCCS	181.5	1.62
MetCCS	175.9	4.66
DeepCCS	168.8	8.51
ISiCLE	194.5	5.42

Supplementary Figure 6.

The comparison of predicted CCS values for three compounds obtained from AllCCS and other tools.

The predicted CCS values and relative errors obtained from AllCCS and other tools: (**a**) gamma-Glutamylglutamate; (**b**) berberine; (**c**) (R)-Glabridin.

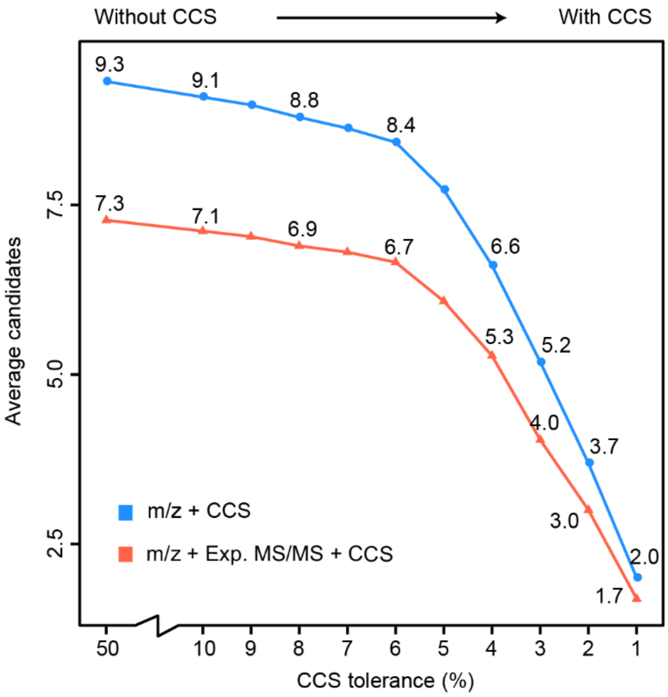

Supplementary Figure 7.

The candidate reduction of the multi-dimensional match using the predicted CCS values and the experimental MS/MS spectral library. The experimental MS/MS spectral library was downloaded from GNPS with a total of 13,499 compounds. Other parameters were kept the same as the in-silico MS/MS spectral match in Figure 4.

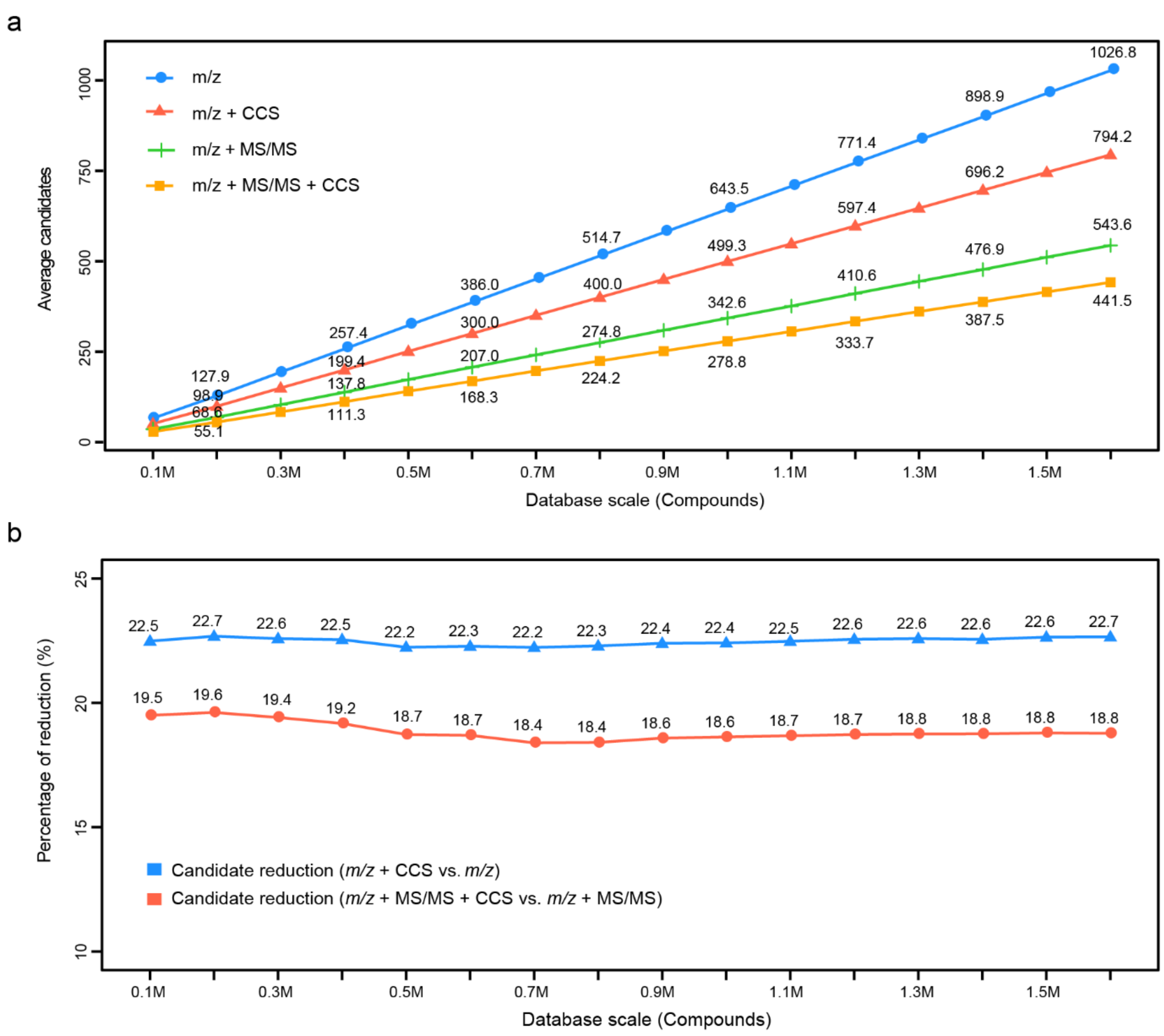

Supplementary Figure 8.

The statistics of average candidates and reduction percentages with the addition of CCS match in different database scales.

(**a**) The statistics of average candidate number through adding the CCS match with different scaled databases; (**b**) the percentage of candidate reduction using different scaled databases. The different scaled databases were randomly sampled from the AllCCS database. The data from the validation set 2 was used.

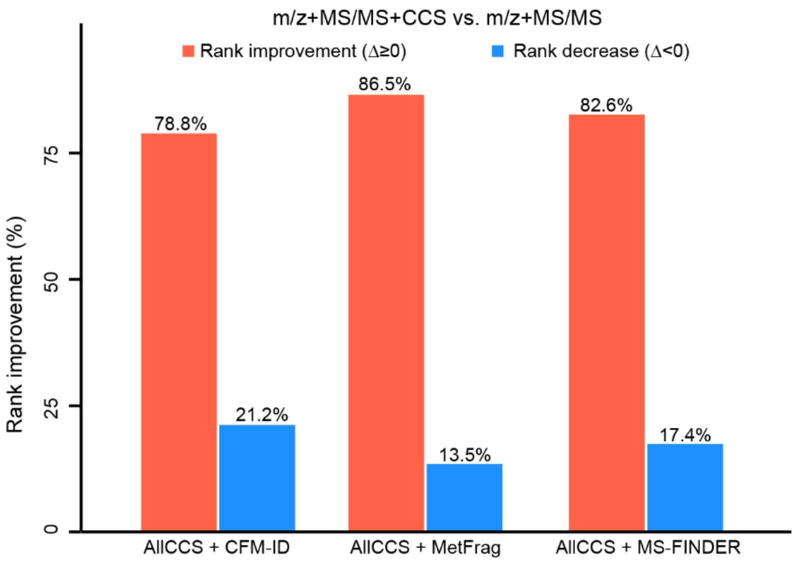

Supplementary Figure 9.

The percentages of rank improvement for correct candidates with the addition of CCS match to the multi-dimensional match in negative ionization mode. Three in-silico MS/MS prediction tools (CFM-ID, MetFrag, and MS-FINDER) were used to generate the predicted MS/MS spectra.

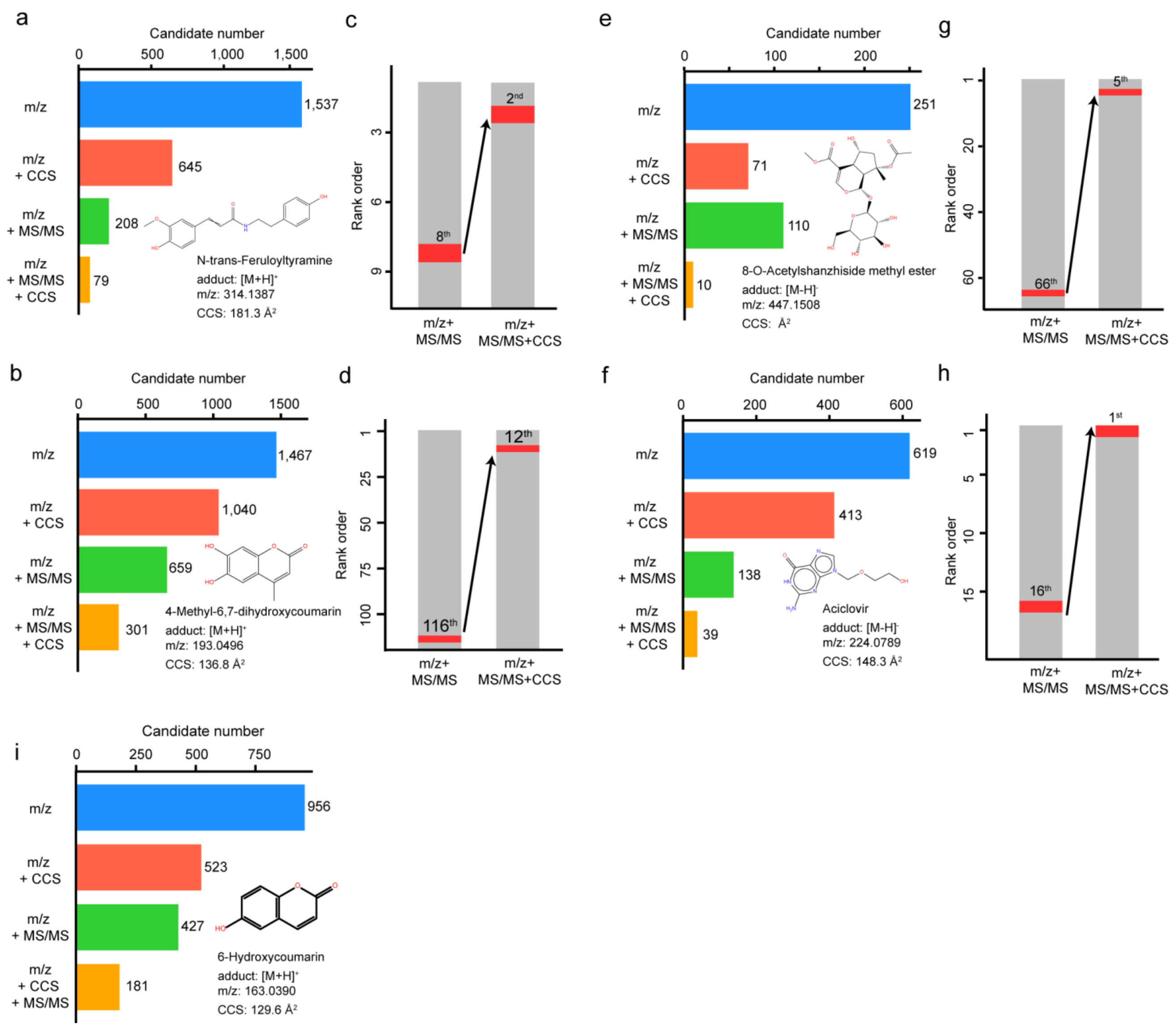

Supplementary Figure 10.

Compound examples for demonstrating the use of AllCCS to improve known metabolite annotations.

(**a-b, e-f**) The candidate reductions for N-trans-Feruloyltyramine (**a**), 4-Methyl-6,7-dihydroxycoumarin (**b**) in positive mode, and 8-O-Acetylshanzhiside methyl ester (**e**), Aciclovir (**f**) in negative mode; (**c-d, g-h**) the rank improvements for N-trans-Feruloyltyramine (**c**), 4-Methyl-6,7-dihydroxycoumarin (**d**) in positive mode, 8-O-Acetylshanzhiside methyl ester (**g**), Aciclovir (**h**) in negative mode; (**i**) the candidate reduction of 6-Hydroxycoumarin (supplementary information for Figure 4c). The in-silico MS/MS tool CFM-ID was used in panels a, c, e, and g. The in-silico MS/MS tool MetFrag was used in panels b, d, f, and h. The in-silico MS/MS tool MS-FINDER was used in panel i.

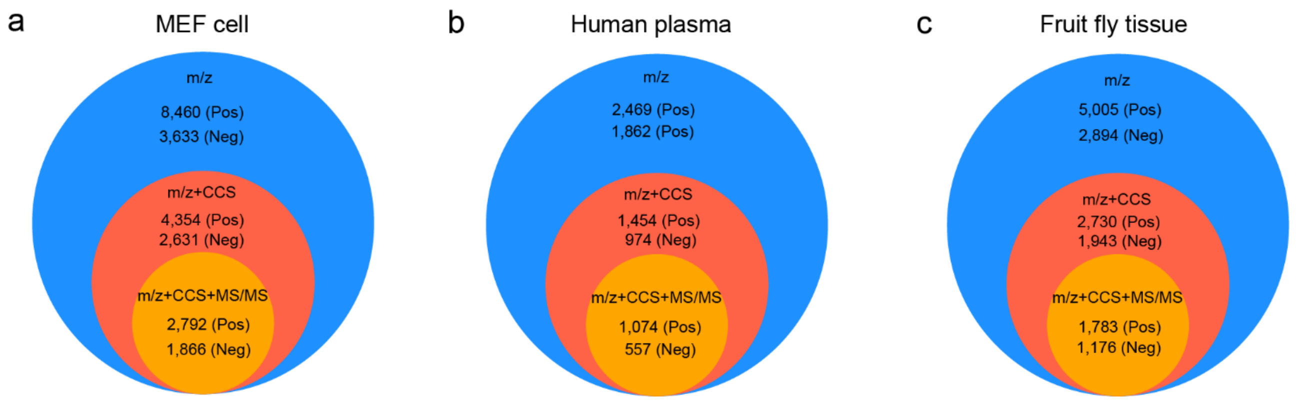

Supplementary Figure 11.

Metabolite annotation with multi-dimensional match for different biological samples.

(**a**) mouse embryonic fibroblast (MEF) cell; (**b**) human plasma; (**c**) fruit fly head tissue. The abbreviations “Pos” and “Neg” represent positive and negative modes, respectively. The KEGG and HMDB databases were used.

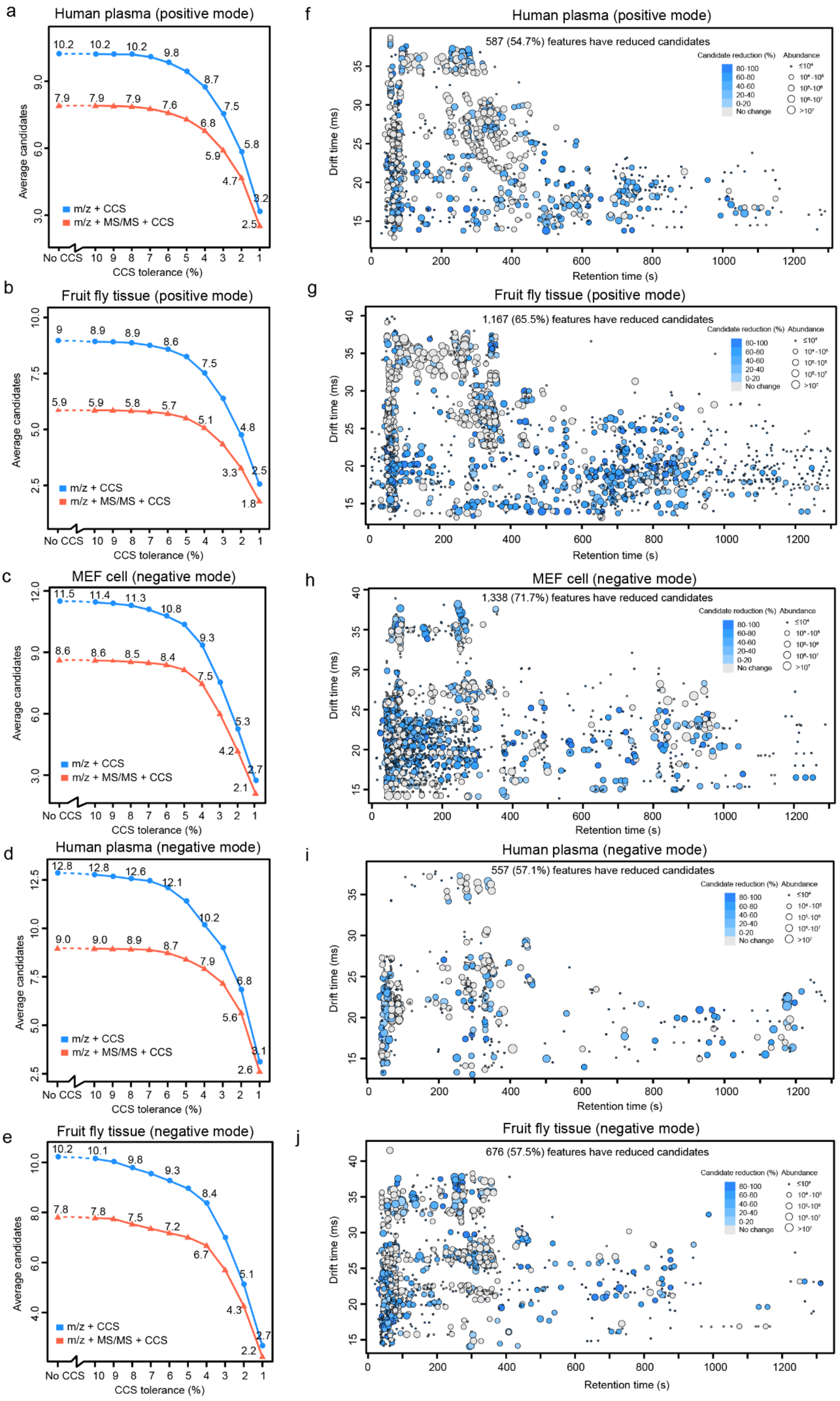
a
Human plasma (positive mode)
10.2 10.2 10.2 9.8 8.7 7.5 5.8 3.2
7.9 7.9 7.9 7.6 6.8 5.9 4.7 2.5
m/z + CCS
m/z + MS/MS + CCS
Average candidates
CCS tolerance (%)
No CCS 10 9 8 7 6 5 4 3 2 1
b
Fruit fly tissue (positive mode)
9 8.9 8.9 8.6 7.5 4.8 2.5
5.9 5.9 5.8 5.7 5.1 3.3 1.8
m/z + CCS
m/z + MS/MS + CCS
Average candidates
CCS tolerance (%)
c
MEF cell (negative mode)
11.5 11.4 11.3 10.8 9.3 5.3 2.7
8.6 8.6 8.5 8.4 7.5 4.2 2.1
m/z + CCS
m/z + MS/MS + CCS
Average candidates
CCS tolerance (%)
d
Human plasma (negative mode)
12.8 12.8 12.6 12.1 10.2 6.8 3.1
9.0 9.0 8.9 8.7 7.9 5.6 2.6
m/z + CCS
m/z + MS/MS + CCS
Average candidates
CCS tolerance (%)
e
Fruit fly tissue (negative mode)
10.2 10.1 9.8 9.3 8.4 5.1 2.7
7.8 7.8 7.5 7.2 6.7 4.3 2.2
m/z + CCS
m/z + MS/MS + CCS
Average candidates
CCS tolerance (%)
f
Human plasma (positive mode)
587 (54.7%) features have reduced candidates
g
Fruit fly tissue (positive mode)
1,167 (65.5%) features have reduced candidates
h
MEF cell (negative mode)
1,338 (71.7%) features have reduced candidates
i
Human plasma (negative mode)
557 (57.1%) features have reduced candidates
j
Fruit fly tissue (negative mode)
676 (57.5%) features have reduced candidates
Candidate reduction (%)
80-100
60-80
40-60
20-40
0-20
No change
Abundance
Drift time (ms)
Retention time (s)

Supplementary Figure 12.

Multi-dimensional match reduces false candidates for different biological samples.

(**a-e**) The reduction of candidate number with the addition of CCS match in human plasma (**a,** positive mode), fruit fly head tissue (**b,** positive mode), MEF cell (**c**, negative mode), human plasma (**d**, negative mode), and fruit fly head tissue (**e**, negative mode); (**f-j**) the percentage of candidate reduction with multi-dimensional match for each feature in human plasma (**f,** positive mode), fruit fly head tissue (**g,** positive mode), MEF cell (**h**, negative mode), human plasma (**i**, negative mode), and fruit fly head tissue (**j**, negative mode).

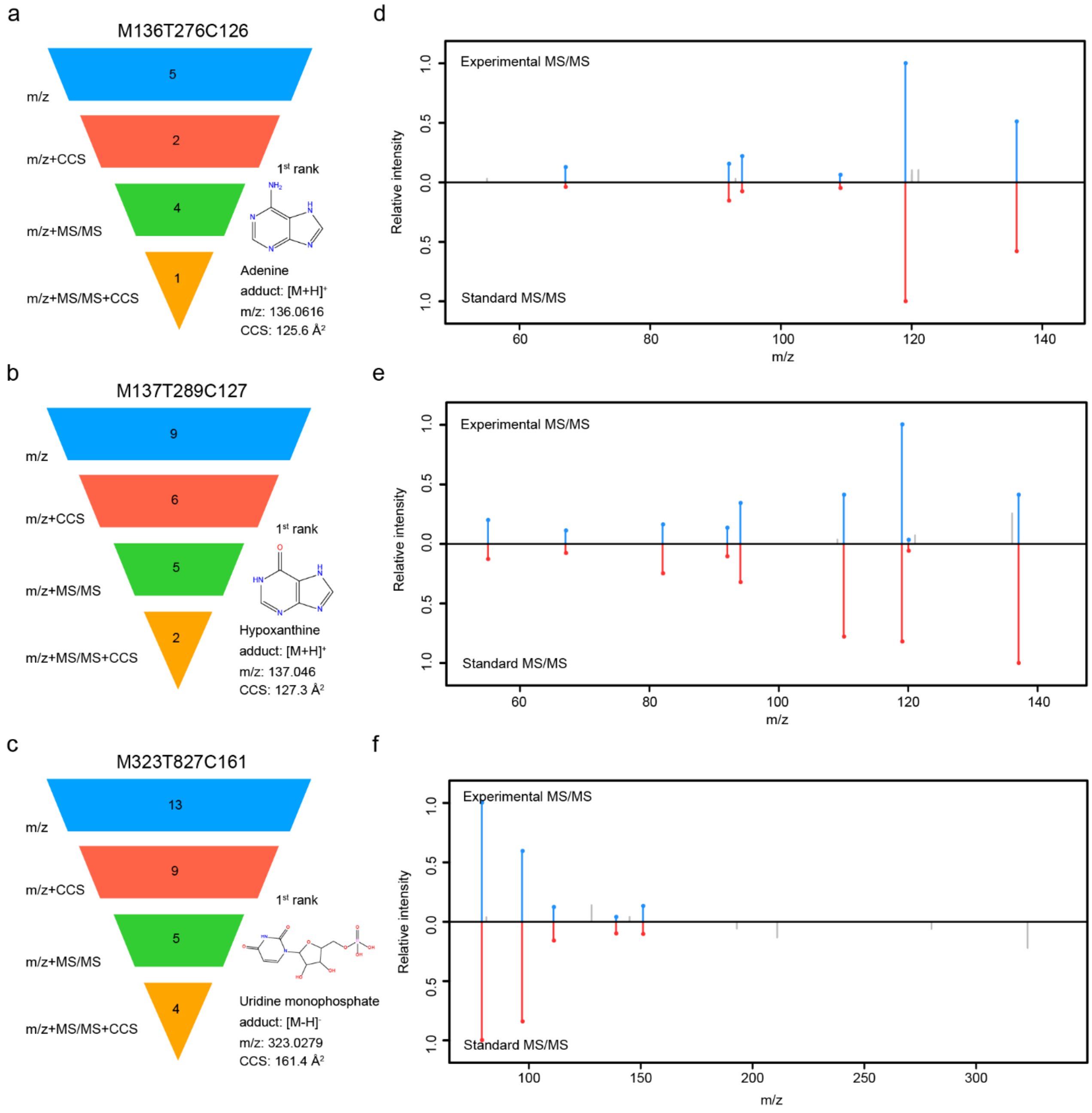

Supplementary Figure 13.

Examples of known metabolite annotations with multi-dimensional match in biological samples.

(**a-c**) The metabolites of adenine (**a**), hypoxanthine (**b**), uridine monophosphate (**c**) were successfully annotated with multi-dimensional match in MEF cell samples (positive mode), fruit fly tissue samples (positive mode), and MEF cell samples (negative moe); (**d-f**) the annotation of adenine (**d**), hypoxanthine (**e**), uridine monophosphate (**f**) were confirmed using chemical standards through matching standard MS/MS spectra (**d-f**) and retention times (less than 15 seconds).

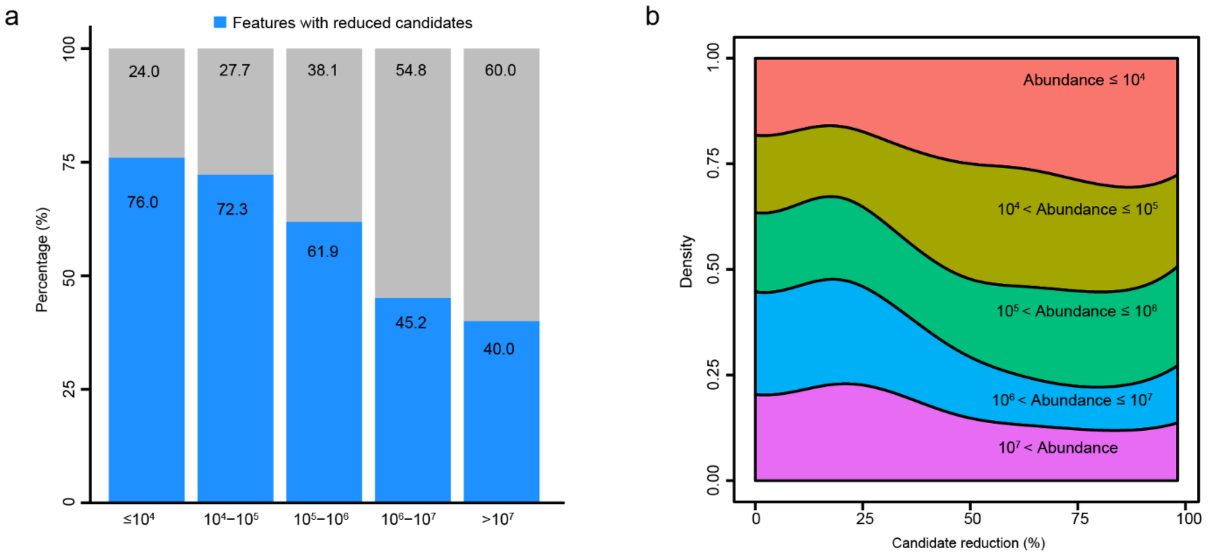

Supplementary Figure 14.

Candidate reduction for features with different abundances.

(**a**) The percentages of reduced candidates for different abundant features; (**b**) the distribution of candidate reduction among different abundant features.

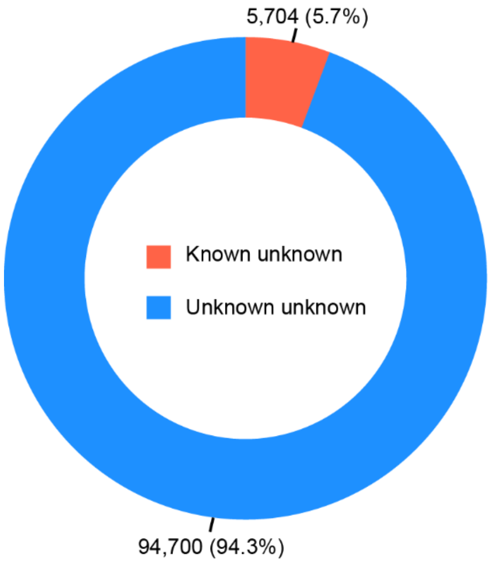

Supplementary Figure 15.

The known unknown and unknown unknown metabolites in extended database.

The known unknown is defined as the structure included in PubChem but not in KEGG; the unknown unknown is defined as the structure not included in either PubChem or KEGG. The InChIKeys of structures were used for searching with KEGG and PubChem.

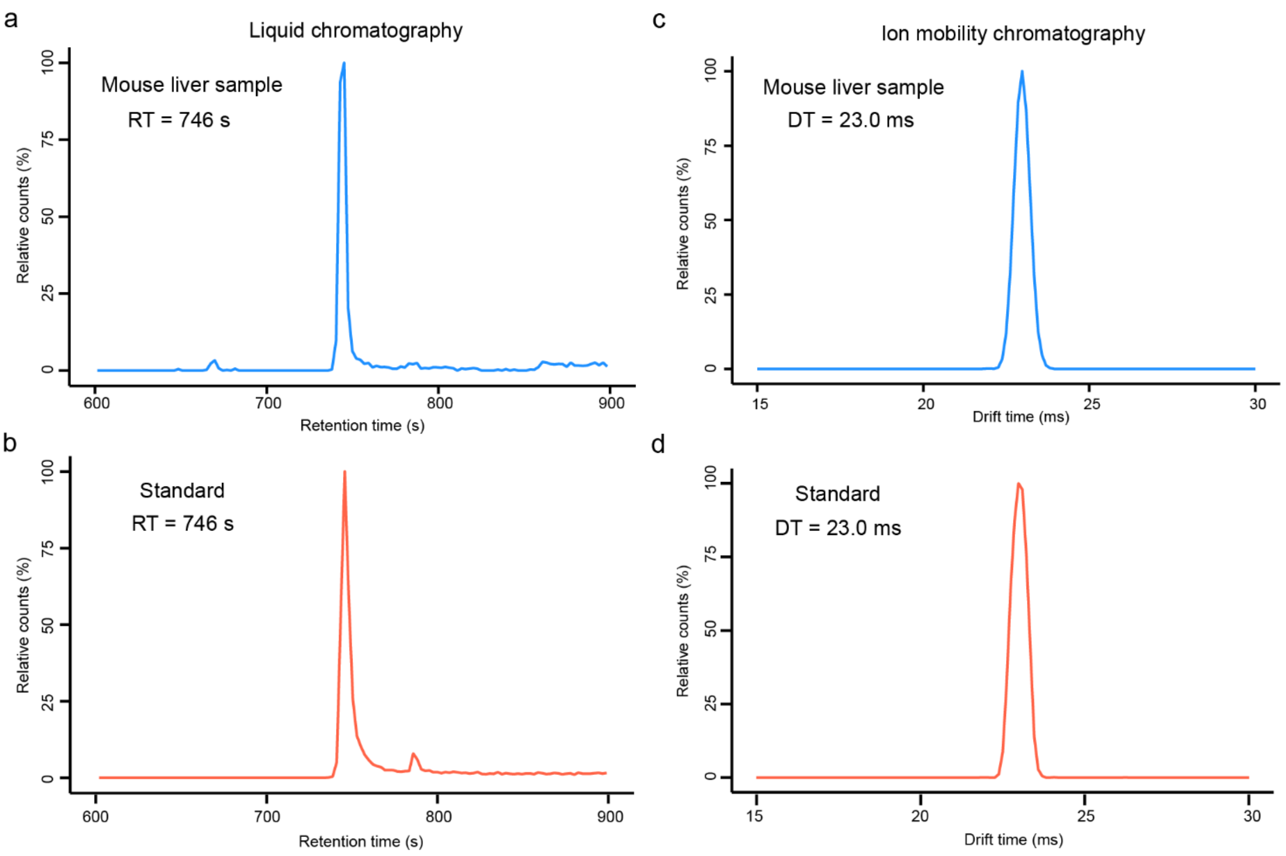

Supplementary Figure 16.

The confirmation of identification of ExtDB016054 using synthesized chemical standard.

(**a-b**) The comparison of retention times obtained from the metabolite in mouse liver sample (**a**) to the chemical standard (**b**); (**c-d**) the comparison of the drift times obtained from the metabolite in mouse liver sample (**c**) to the chemical standard (**d**).

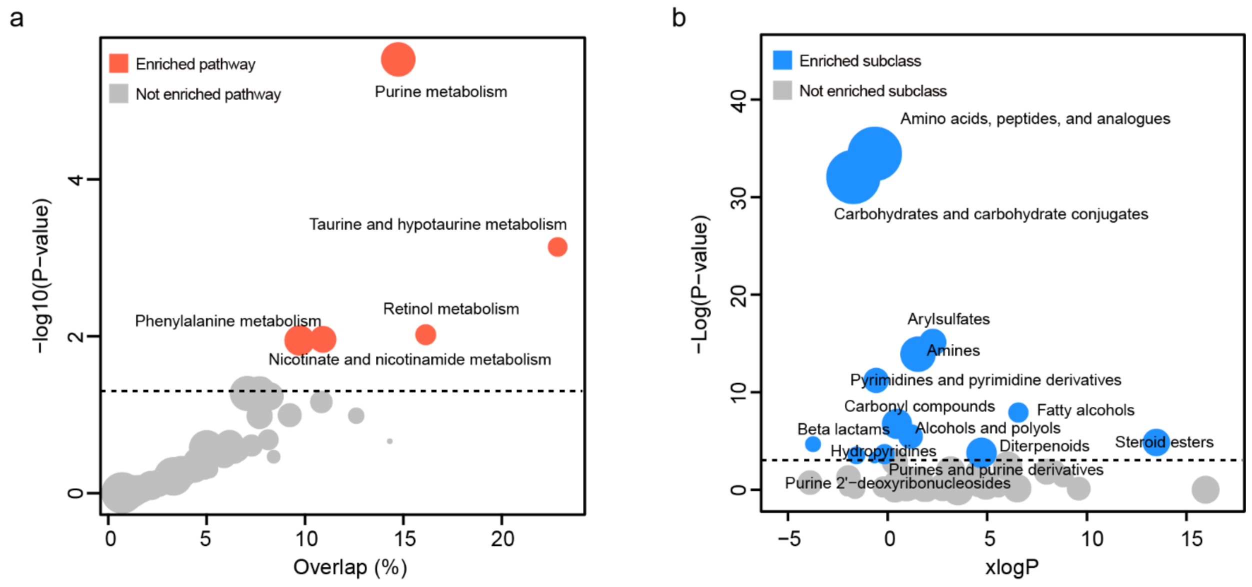

Supplementary Figure 17.

The pathway and structure enrichment analyses.

(**a**) Pathway enrichment analysis of KEGG metabolites (36-week vs. 104-week; p-value≤0.05; two-sided hypergeometric test); The dot size represents the metabolite numbers in the pathway; (**b**) chemical subclass enrichment analysis of unknown metabolites (p-value≤0.05; two-sided Kolmogorov-Smirnov test in ChemRich[1]); The dot size represents the metabolite numbers in the subclass; The chemical classes of metabolites were obtained from ClassyFire[2].

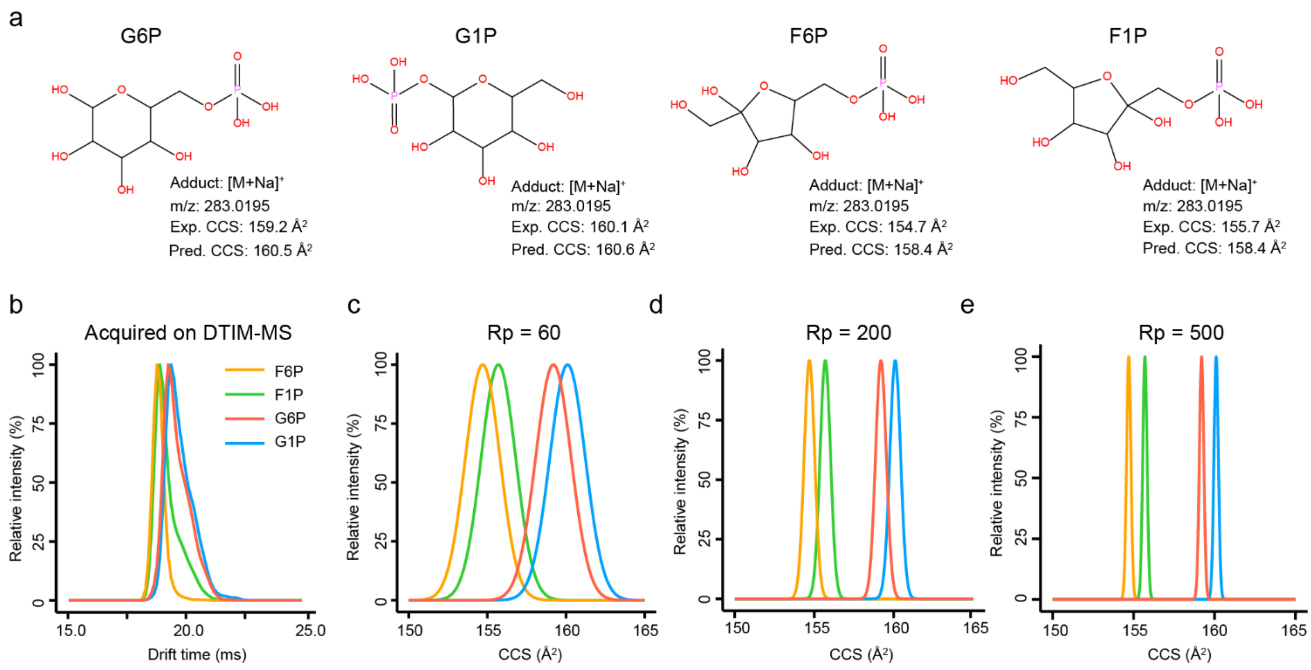

Supplementary Figure 18.

The IM separation for 4 monosaccharide phosphate isomers.

(**a**) The structures of 4 monosaccharide phosphate isomers: glucose-6-phosphate, G6P; glucose-1-phosphate, G1P; fructose-6-phosphate, F6P; fructose-1-phosphate, F6P; (**b**) the separation of 4 monosaccharide phosphate isomers with Agilent DTIM-MS 6560 with a IM resolution of 40-60; (**c-e**) the simulated IM separations of monosaccharide phosphate isomers using different IM resolving powers (Rp): Rp=60 (**c**); Rp=200 (**d**); and Rp=500 Rp (**e**). Rp was defined as the ion drift time (DT) divided by the peak full width at half-maximum height (FWHM) in DTIM-MS[3].

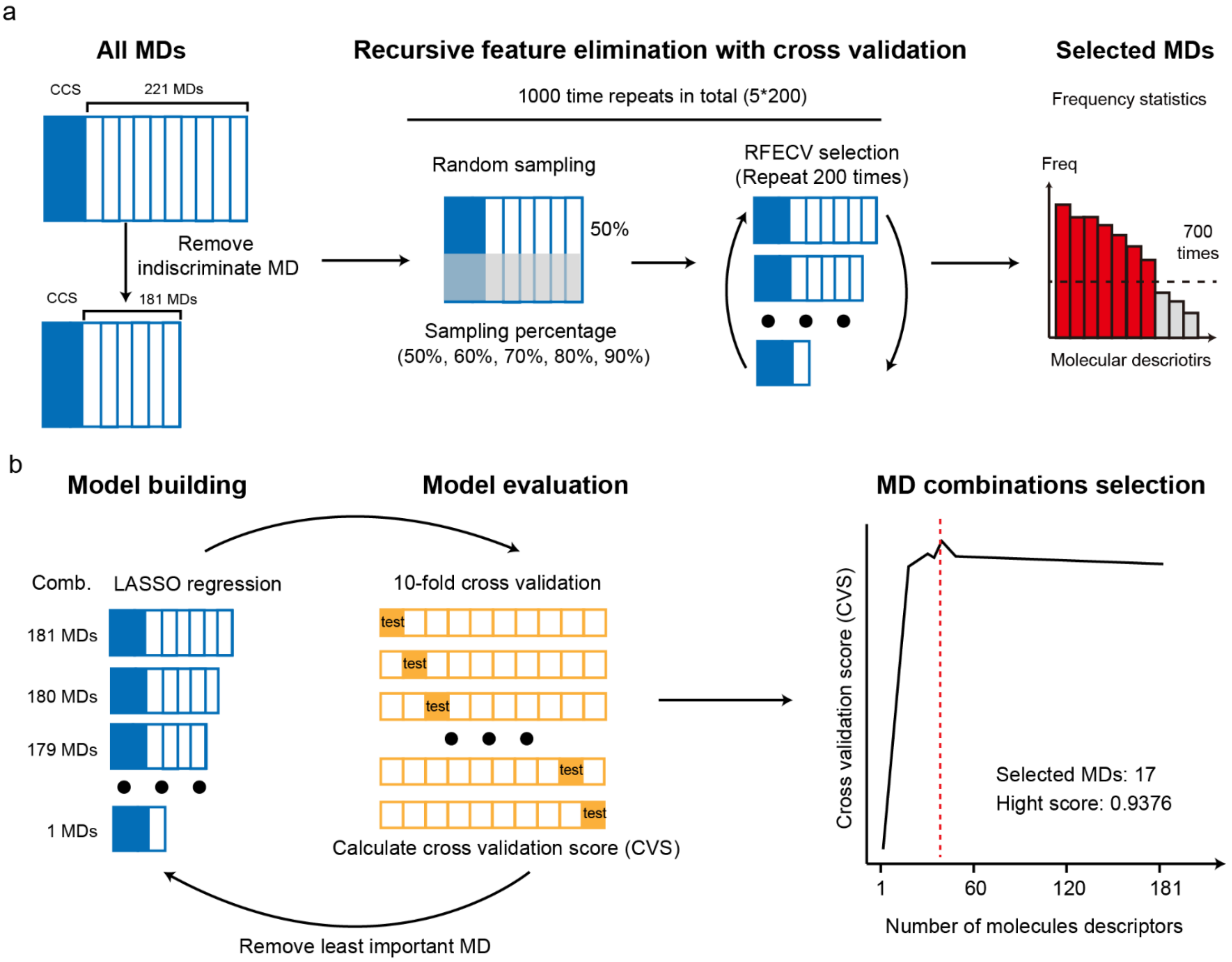

Supplementary Figure 19.

Schematic illustration of molecular descriptor (MD) selection.

(**a**) The general workflow of molecule descriptor selection; (**b**) the detailed procedures for recursive feature elimination with cross validation (RFECV).

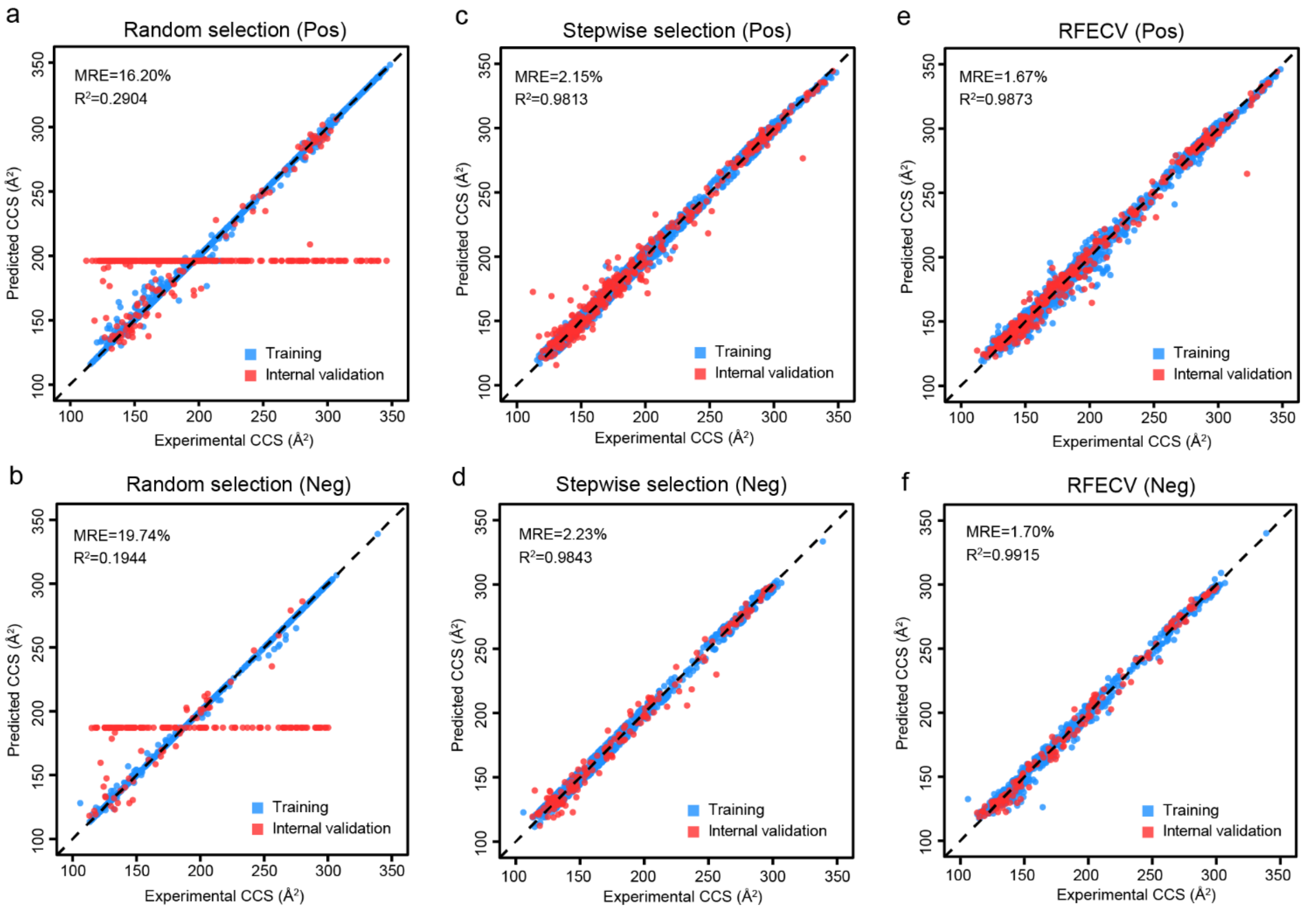

Supplementary Figure 20.

The comparison of different molecular descriptor selection approaches.

(**a-b**) Random selection of molecular descriptors in positive (**a**) and negative modes (**b**); (**c-d**) stepwise selection of molecular descriptors in positive (**c**) and negative modes (**d**); (**e-f**) recursive feature elimination with cross validation (RFECV) for molecular descriptor selection in positive (**e**) and negative modes (**f**).

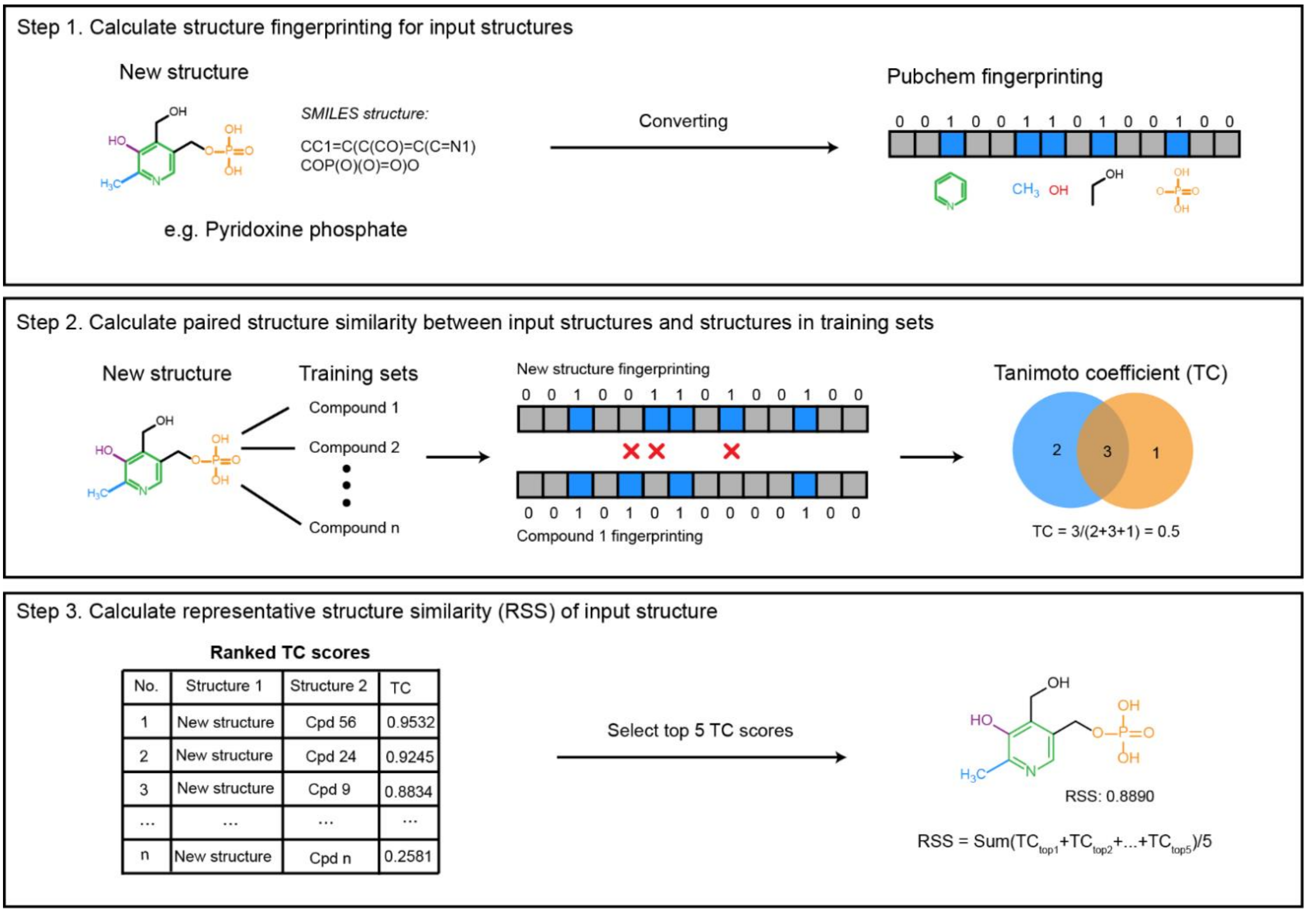

Supplementary Figure 21.

Schematic illustration of representative structure similarity (RSS) calculation.

Molecular fingerprinting (PubChem format) of each input new structure is first calculated (step 1). Then, AllCCS calculates the paired structure similarity between input structure and one structure in the training set using molecular fingerprinting and the tanimoto coefficient (step 2). Finally, representative structure similarity is calculated as the mean value of top 5 ranked TC scores (step 3).

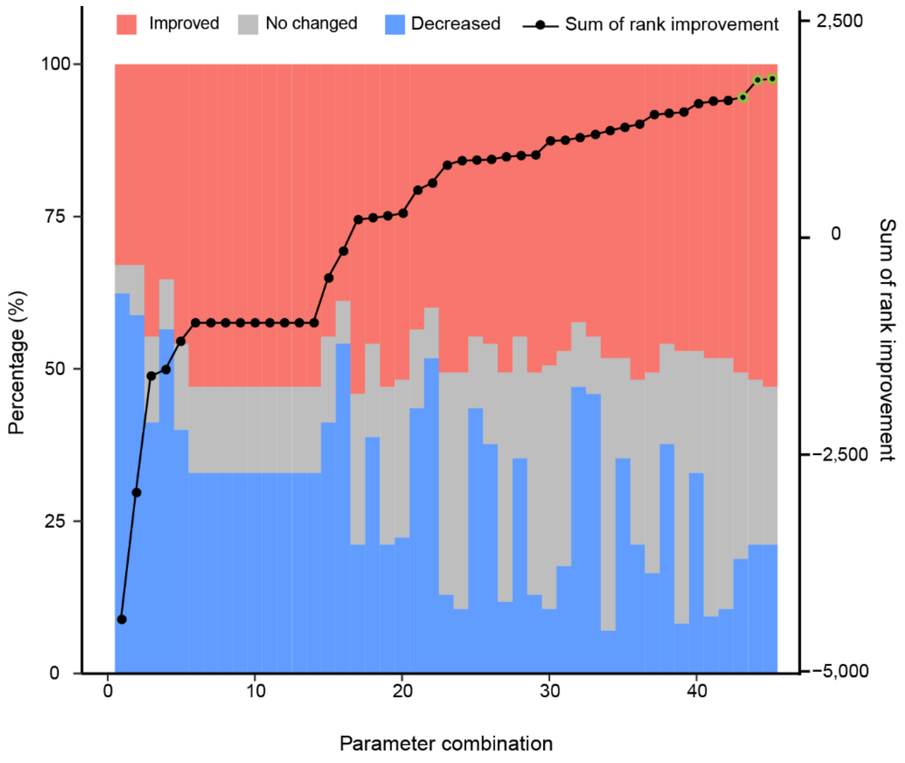

Supplementary Figure 22.

The optimization of score weight and tolerance of CCS match in multi-dimensional match.

45 parameter combinations for different minimum tolerances, weights of CCS match score and weights of MS/MS match score were optimized. The bar plot is the percentage of rank changes with each parameter combination. Each black dots represents the sum of rank improvement with each parameter combination. The range of best parameter combination (black dots with green circles): minimum CCS tolerance, 2%; weight of MS/MS match score, 0.5-0.7; weight of CCS match score, 0.5-0.3. In this work, we used the following parameters for scoring in multi-dimensional match unless otherwise specified: minimum CCS tolerance, 2%; maximum CCS tolerance, 4%; weight of CCS score, 0.3; weight of MS/MS score, 0.7.

Supplementary Table 1.

The collection and standardization of experimental CCS records from publications.

No.	Lab	Literature	Instrument (Polarity, Approach)	Reported CCS records	Included in AllCCS
1	Zhu Lab	Zhou et. al.[4]	DTIMS (Pos, SF)	511	420
2	Zhu Lab	Zhou et. al.[4]	DTIMS (Neg, SF)	615	503
3	Zhu Lab	Zhou et. al.[5]	DTIMS (Pos, SF)	411	411
4	Zhu Lab	Zhou et. al.[5]	DTIMS (Neg, SF)	161	161
5	Xu Lab	Hines et. al.[6]	TWIMS (Pos, EM)	154	143
6	Xu Lab	Hines et. al.[6]	TWIMS (Neg, EM)	92	83
7	Xu Lab	Hines et. al. [7]	TWIMS (Pos, EM)	1,570	876
8	Xu Lab	Hines et. al. [7]	TWIMS (Neg, EM)	6	1
9	Baker Lab	Zheng et. al.[8]	DTIMS (Pos, MF)	733	566
10	Baker Lab	Zheng et. al.[8]	DTIMS (Neg, MF)	402	321
11	McLean Lab	Nichols et. al.[9]	DTIMS (Pos, MF)	550	450
12	McLean Lab	Nichols et. al.[9]	DTIMS (Neg, MF)	431	362
13	McLean Lab	Nichols et. al.[9]	DTIMS (Pos, SF)	548	455
14	McLean Lab	Nichols et. al.[9]	DTIMS (Neg, SF)	432	367
		**Sum**		6,616	5,119

Note: The abbreviations of “Pos” and “Neg” represent positive and negative ionization modes, respectively; the abbreviation of “SF”, “MF” and “EM” represent the single-field, the multiple-field and empirical method, respectively.

Supplementary Table 2.

The definition of confidence levels for CCS values in AllCCS.

Confidence level	Platform	Reported labs (N)	Maximum relative error (%)	No. of compounds	No. of CCS values
Level 1	DTIMS	N≥2	≤1%	272	462
Level 2	DTIMS/TWIMS/TIMS	N≥2	≤3%	349	448
Level 3	DTIMS/TWIMS/TIMS	N=1	---	1,865	2,491
Level 4	Predicted CCS	---	---	1,670,596	11,694,172
Conflict	DTIMS/TWIMS/TIMS	N≥2	>3%	119	138

Supplementary Table 3.

The statistics of ion adducts in the unified CCS database.

Positive mode		Negative mode	
**Adduct**	**Number**	**Adduct**	**Number**
$[M+H]^+$	1,475	$[M-H]^-$	885
$[M+Na]^+$	701	$[M+Na-2H]^-$	146
$[M+NH_4]^+$	134	$[M+HCOO]^-$	49
$[M+H-H2O]^+$	56	$[M-H_2O-H]^-$	25
$[M+K]^+$	27	$[M+CH_3COO]^-$	9
$[M-H+2Na]^+$	17	$[M+K-2H]^-$	2
$[M]^+$	10		
$[M-H+2K]^+$	2		
$[M-2H+3Na]^+$	1		
Sum	2,423	Sum	1,116

Supplementary Table 4.

The statistics of super classes of compounds in the unified CCS database.

No.	Superclass	Compounds (CCS values)	m/z range	CCS range (Å^2)
1	Lipids and lipid-like molecules	707 (1,229)	[115.0764, 1034.3835]	[118.6, 348.5]
2	Organoheterocyclic compounds	365 (505)	[95.0604, 1377.5567]	[114.0, 340.1]
3	Benzenoids	307 (359)	[108.0455, 559.2300]	[108.8, 226.8]
4	Organic acids and derivatives	253 (502)	[102.0560, 1005.4292]	[105.9, 299.0]
5	Organic oxygen compounds	196 (335)	[105.0193, 1071.3733]	[113.3, 317.6]
6	Phenylpropanoids and polyketides	149 (199)	[133.0648, 936.5444]	[122.2, 311.6]
7	Nucleosides, nucleotides, and analogues	107 (287)	[226.0833, 835.0410]	[148.0, 256.1]
8	Organic nitrogen compounds	39 (49)	[104.1070, 505.3246]	[117.7, 223.9]
9	Alkaloids and derivatives	29 (30)	[138.0550, 811.4277]	[124.9, 278.5]
10	Lignans, neolignans and related compounds	6 (6)	[355.1176, 679.1457]	[171.0, 266.1]
11	Hydrocarbon derivatives	2 (2)	[344.1493, 400.1755]	[181.0, 194.5]
12	Organometallic compounds	2 (3)	[138.9735, 270.9224]	[112.5, 139.2]
13	Organosulfur compounds	2 (3)	[179.0018, 237.0781]	[126.9, 171.3]
14	Homogeneous non-metal compounds	1 (1)	[200.9325, 200.9325]	[145.6, 145.6]
15	Others (Not defined superclass)	28 (29)	[103.0400, 985.5003]	[118.2, 322.5]
**Summary**		**2,193 (3,539)**	**[95.0604, 1377.5567]**	**[114.0, 348.5]**

Supplementary Table 5.

Performance evaluation using the external validation sets 1 and 2.

Validation sets	Ion polarity	No. of CCS values	Mean relative error (%)	Median relative error (%)	R^2
Validation set 1 (metabolites and lipids)	Positive mode	463	2.26	1.66	0.9901
Validation set 1 (metabolites and lipids)	Negative mode	199	2.22	1.74	0.9850
Validation set 2 (drugs and natural products)	Positive mode	107	2.50	1.81	0.9687
Validation set 2 (drugs and natural products)	Negative mode	122	3.79	2.25	0.9230

Supplementary Table 6.

The comparison of different CCS calculation tools.

	**AllCCS**	**MetCCS[4,10]**	**DeepCCS[11]**	**ISiCLE[12]**
**Implementation**	Web server	Web server	Command line	Command line
**CCS calculation (Open to use)**	√	√	√	x
**CCS Database**	√	√	x	√
**Time consuming (Per compound)**	1-10 s	~5 min	1-10 s	37.2 node-hours
**Prediction error (Median relative error)**	1-2%	2-3%	2-3%	~5% (Lite)<br>~3% (Standard)
**Compounds**	~1,700,000	~35,000	x	~700,000
**Experimental CCS**	5,119	x	x	1,454
**In-silico CCS**	~12,000,000	~170,000	x	~2,000,000
**Compound coverage**	Metabolites<br>Lipids<br>Natural product<br>Drugs<br>Toxins	Metabolites	Metabolites	Endogenous Small molecule<br>Natural product<br>Toxins
**Ease of use**	Easy	Medium	Difficult	Difficult

Supplementary Table 7.

The training set comparison for machine-learning based CCS prediction tools

**Tools**	**AllCCS**	**MetCCS**	**DeepCCS**
No. of compounds	1,873	514	1,076
No. of CCS values	2,646	796	1,665
No. of classes	131	52	104
No. of source labs	4	1	4
CCS standardization	Yes	No	No
MD optimization	Yes	No	Not applicable

Note: ISiCLE was excluded from the comparison because it uses a quantum-chemistry based approach for CCS calculation.

Supplementary Table 8.

The possible ion adducts in the data cleaning of experimental CCS values.

**No.**	**Positive mode**		**Negative mode**	
	Adduct	m/z difference	Adduct	m/z difference
1	$[M]^+$	-0.0005	$[M]^-$	0.0005
2	$[M+H]^+$	1.0073	$[M-H]^-$	-1.0073
3	$[M+H-H_2O]^+$	-17.0033	$[M-H_2O-H]^-$	-19.0179
4	$[M+H-2H_2O]^+$	-35.0139	$[M+Na-2H]^-$	20.9747
5	$[M+NH_4]^+$	18.0339	$[M+K-2H]^-$	36.9486
6	$[M+Na]^+$	22.9893	$[M+NH_4-2H]^-$	16.0193
7	$[M-H+2Na]^+$	44.9713	$[2M-H]^-$	-1.0073
8	$[M-2H+3Na]^+$	66.9533	$[M+CH_3COO]^-$	59.0138
9	$[M+K]^+$	38.9632	$[M+F]^-$	18.9989
10	$[M-H+2K]^+$	76.9191	$[M+HCOO]^-$	44.9982
11	$[M-2H+3K]^+$	114.8750		
12	$[M+CH_3CN+H]^+$	42.0038		
13	$[M+CH_3CN+Na]^+$	64.0158		
14	$[2M+H]^+$	1.0073		
15	$[2M+NH_4]^+$	18.0339		
16	$[2M+Na]^+$	22.9893		
17	$[2M+K]^+$	38.9632		
18	$[M+CH_3COO+2H]^+$	61.0284		
19	$[M+HCOO+2H]^+$	47.0128		

Supplementary Table 9.

The selected molecular descriptors in positive and negative modes.

Positive mode			Negative mode		
No.	Name	Frequency	No.	Name	Frequency
1	mz	1,000	1	mz	1,000
2	VAdjMat	1,000	2	VAdjMat	1,000
3	FMF	999	3	nAtomLAC	999
4	nAtomLAC	998	4	VP.0	996
5	khs.ssCH2	997	5	ALogp2	986
6	Kier3	995	6	nAtom	981
7	nAtom	993	7	VP.1	976
8	VP.0	965	8	nAtomLC	963
9	C2SP3	961	9	Khs.ssssN	959
10	VP1	941			
11	MDEC.12	862			
12	ALogp2	860			
13	VP.2	843			
14	nAtomLC	802			
15	AMR	724			

Note: please refer the references 12 and 13 for the detailed descriptions for each molecular descriptor[13,14].

Supplementary Table 10.

The comparison of different molecular descriptor selection approaches

Validation	Ion polarity	Number of MDs	Mean relative error (%)	Median relative error (%)	R^2
Random selection	Positive	78	20.04	16.20	0.2904
Random selection	Negative	64	20.33	19.74	0.1944
Stepwise selection	Positive	78	3.04	2.15	0.9813
Stepwise selection	Negative	64	3.02	2.23	0.9843
RFECV	Positive	15	2.45	1.67	0.9873
RFECV	Negative	9	2.24	1.70	0.9915

Supplementary Table 11.

Performance evaluation of the prediction model.

Ion polarity	Data set size	Mean relative error (%)	Median relative error (%)	R^2	q^2
Positive	1851 CCS values	2.21	1.67	0.9913	0.9899
Negative	795 CCS values	2.11	1.72	0.9928	0.9903

Note: q^2 is mean value of R^2 by 10 times 10-folds cross validation; R^2 values were calculated by Pearson correlation.

Supplementary Table 12.

The comparison between SVR and MLR.

	**Validation set 1**		**Validation set 2**	
	**SVR**	**MLR**	**SVR**	**MLR**
Median relative error (%)	1.67	1.82	2.03	2.94
Mean relative error (%)	2.25	2.60	3.19	3.44
Percentages (error ≤ 4%)	85.6	77.5	76.4	66.3

Note: the abbreviations SVR and MLR represent support vector regression and multiple linear regression, respectively.

Supplementary Table 13.

The information of compounds in predicted AllCCS database.

No.	Database	Compounds	Coverage	Mirror date	Reference
1	KEGG	16,085	Metabolites & lipids	2018-08-02	Kanehisa et. al.[15]
2	HMDB	113,989	Metabolites & lipids	2018-06-09	Wishart et. al.[16]
3	LMSD	40,532	Metabolites & lipids	2019-07-11	Fahy et. al.[17]
4	MINE	592,175	Metabolites & lipids	2018-02-07	Jeffryes et. al.[18]
5	DrugBank	9,546	Drugs & xenobiolics	2019-04-12	Wishart et. al.[19]
6	DSSTox	856,919	Drugs & xenobiolics	2019-05-06	Grulke et. al.[20]
7	UNPD	213,188	Natural products	2019-06-13	Gu et. al.[21]

Supplementary Table 14.

The parameters for the in-silico MS/MS tools.

In-silico MS/MS	Version	Parameter
MetFrag	2.4.5-CL	IsPositiveIonMode = True
		DatabaseSearchRelativeMassDeviation = 25
		FragmentPeakMatchAbsoluteMassDeviation = 0.005
		FragmentPeakMatchRelativeMassDeviation = 25
		MaximumTreeDepth = 2
		NumberThreads = 4
		MetFragCandidateWriter = CSV
		MetFragDatabaseType = LocalCSV
		MetFragPreProcessingCandidateFilter =
		UnconnectedCompoundFilter,IsotopeFilter
		MetFragScoreWeights = 1.0
		MetFragScoreTypes = FragmenterScore
CFM-ID	2.4	num_highest = -1
		ppm_mass_tol = 10
		abs_mass_tol = 0.01
		prob_thresh = 0.001
		param_file = ' param_output0.log'
		config_file = 'param_config.txt'
		score_type = 'Jaccard'
		apply_postprocessing = 1
MS-FINDER	3.24	#Formula finder parameters
		LewisAndSeniorCheck=True
		Ms1Tolerance=0.005
		IsotopicAbundanceTolerance=20
		MassToleranceType=Da
		CommonRange=TRUE
		ExtendedRange=FALSE
		ExtremeRange=FALSE
		ElementProbabilityCheck=False
		Ocheck=True
		Ncheck=True
		Pcheck=True
		Scheck=True
		Fcheck=True
		ClCheck=True
		BrCheck=True
		Icheck=True
		SiCheck=True
		IsTmsMeoxDerivative=False
		FormulaMaximumReportNumber=100

```
#Structure finder parameters
TreeDepth=2
Ms2Tolerance=0.005
RelativeAbundanceCutOff=1
StructureMaximumReportNumber=100
IsUseEiFragmentDB=False

#Data source
MinesNeverUse=True
MinesOnlyUseForNecessary=False
MinesAllways=False
PubChemNeverUse=True
PubChemOnlyUseForNecessary=False
IsUserDefinedDB=True

#Spectral database search
IsRunSpectralDbSearch=False
IsRunInSilicoFragmenterSearch=True
IsPrecursorOrientedSearch=True
MassRangeMin=50
MassRangeMax=1000

#Batch job
AllProcess=True
FormulaFinder=True
StructureFinder=True
TryTopNMolecularFormulaSearch=5
```

Supplementary Table 15.

The parameters for LC-IM-MS/MS data processing.

**Software**	**Version**	**Parameters**
PNNL PreProcessor	V2018.06.02	Moving average smoothing number of points (checked)<br>m/z: none<br>drift: 3<br>chromatography/infusion: 3<br>signal intensity lower threshold (counts): 20<br>Repair saturation for points above abundance limit (%): checked, 40<br>Repair fragments spectra (high CE): checked
Agilent Mass Profiler	V10.0	# Feature Finding/Loading<br>Measure of abundance: Max ion volume<br>Feature finding input filters: Chromatographic data<br>Isotope mode: Common organic molecules<br>Limit charge states to range: 1-1<br><br># Alignment & Normalization<br>RT tolerance: 0.0% + 0.3 min<br>DT tolerance: 1.5%<br>Mass: 15.0 ppm + 2.0 mDa<br><br># Statistics & Filters<br>Missing sample treatment: Assign 0 abundance<br>Feature filter: Q-score>70.0<br>Sample occurrence: Frequency ≥ 50% in at least one group<br><br># .CEF Export Options<br>Limit to the largest 10000 compounds, checked<br>Minimum precursor m/z: 50, checed<br>RT-extraction window width (±): 0.0 sec<br>DT-extraction window width (±): 0.5 ms<br>Limit (by height) to the largest: 100 peaks, checked<br>Absolute height: 100.0 counts<br><br># Fragment spectrum drift time offset<br>Absolute offset: -0.3 ms<br>Relative offset: 0.0%

Supplementary references:

1. Barupal, D. K. & Fiehn, O. Chemical Similarity Enrichment Analysis (ChemRICH) as alternative to biochemical pathway mapping for metabolomic datasets. *Sci. Rep.* **7**, 1–11 (2017).
2. Djoumbou Feunang, Y. *et al.* ClassyFire: automated chemical classification with a comprehensive, computable taxonomy. *J. Cheminform.* **8**, 61 (2016).
3. Dodds, J. N., May, J. C. & McLean, J. A. Investigation of the Complete Suite of the Leucine and Isoleucine Isomers: Toward Prediction of Ion Mobility Separation Capabilities. *Anal. Chem.* **89**, 952–959 (2017).
4. Zhou, Z., Shen, X., Tu, J. & Zhu, Z. J. Large-scale prediction of collision cross-section values for metabolites in ion mobility-mass spectrometry. *Anal. Chem.* **88**, 11084–11091 (2016).
5. Zhou, Z., Tu, J., Xiong, X., Shen, X. & Zhu, Z. J. LipidCCS: Prediction of Collision Cross-Section Values for Lipids with High Precision to Support Ion Mobility-Mass Spectrometry-Based Lipidomics. *Anal. Chem.* **89**, 9559–9566 (2017).
6. Hines, K. M., Herron, J. & Xu, L. Assessment of altered lipid homeostasis by HILIC-ion mobility-mass spectrometry-based lipidomics. *J. Lipid Res.* **58**, 809–819 (2017).
7. Hines, K. M., Ross, D. H., Davidson, K. L., Bush, M. F. & Xu, L. Large-Scale Structural Characterization of Drug and Drug-Like Compounds by High-Throughput Ion Mobility-Mass Spectrometry. *Anal. Chem.* **89**, 9023–9030 (2017).
8. Zheng, X. *et al.* A structural examination and collision cross section database for over 500 metabolites and xenobiotics using drift tube ion mobility spectrometry. *Chem. Sci.* **8**, 7724–7736 (2017).
9. Nichols, C. M. *et al.* Untargeted Molecular Discovery in Primary Metabolism: Collision Cross Section as a Molecular Descriptor in Ion Mobility-Mass Spectrometry. *Anal. Chem.* **90**, 14484–14492 (2018).
10. Zhou, Z., Xiong, X. & Zhu, Z.-J. MetCCS predictor: a web server for predicting collision cross-section values of metabolites in ion mobility-mass spectrometry based metabolomics. *Bioinformatics* **33**, 2235–2237 (2017).
11. Plante, P.-L. *et al.* Predicting Ion Mobility Collision Cross-Sections Using a Deep Neural Network: DeepCCS. *Anal. Chem.* **91**, 5191–5199 (2019).
12. Colby, S. M. *et al.* ISiCLE: A Quantum Chemistry Pipeline for Establishing in Silico Collision Cross Section Libraries. *Anal. Chem.* **91**, 4346–4356 (2019).
13. Guha, R. Chemical informatics functionality in R. *J. Stat. Softw.* **18**, 1–16 (2007).
14. CDK manual. Available at: http://docs.ochem.eu/display/MAN/CDK.
15. Kanehisa, M. KEGG: Kyoto Encyclopedia of Genes and Genomes. *Nucleic Acids Res.* **28**, 27–30 (2000).
16. Wishart, D. S. *et al.* HMDB 4.0: the human metabolome database for 2018. *Nucleic Acids Res.* **46**, D608–D617 (2018).
17. Sud, M. *et al.* LMSD: LIPID MAPS structure database. *Nucleic Acids Res* **35**, D527-32 (2007).
18. Jeffryes, J. G. *et al.* MINEs: Open access databases of computationally predicted enzyme promiscuity products for untargeted metabolomics. *J. Cheminform.* **7**, 1–8 (2015).
19. Wishart, D. S. *et al.* DrugBank 5.0: a major update to the DrugBank database for 2018. *Nucleic Acids Res.* **46**, D1074–D1082 (2018).
20. Grulke, C. M., Williams, A. J., Thillanadarajah, I. & Richard, A. M. EPA ' s DSSTox database : History of development of a curated chemistry resource supporting computational toxicology research. *Comput. Toxicol.* **12**, 100096 (2019).
21. Gu, J. *et al.* Use of Natural Products as Chemical Library for Drug Discovery and Network Pharmacology. *PLoS One* **8**, e62839 (2013).
