# Supplementary material for: Ion mobility collision cross-section atlas for known and unknown metabolite annotation in untargeted metabolomics
Source: Nat Commun. 2020 Aug 28;11:4334. doi: 10.1038/s41467-020-18171-8 (PMC7455731; doi:10.1038/s41467-020-18171-8)
Supplement: Supplementary file 3 — Description of Additional Supplementary Files [file 41467_2020_18171_MOESM3_ESM.docx]

**Description of Additional Supplementary Files**

**File Name:** Supplementary Data 1

**Description:** Unified CCS database for model training.

**File Name:** Supplementary Data 2

**Description:** Validation set 1 (metabolites and lipids).

**File Name:** Supplementary Data 3

**Description:** Validation set 2 (drugs and natural products).

**File Name:** Supplementary Data 4

**Description:** Benchmark data for CCS prediction comparison.

**File Name:** Supplementary Data 5

**Description:** In-silico MS/MS spectra of CFM-ID.

**File Name:** Supplementary Data 6

**Description:** Unknowns curated via in-silico reaction.

**File Name:** Supplementary Data 7

**Description:** Known metabolite annotation for different biological samples.

**File Name:** Supplementary Data 8

**Description:** Unknown metabolite annotation for aging mouse samples (36-week vs. 104-week; n=10, biologically independent samples for each group; two-sided Student’s t-test).
