# Supplementary material for: Ion mobility collision cross-section atlas for known and unknown metabolite annotation in untargeted metabolomics
Source: Nat Commun. 2020 Aug 28;11:4334. doi: 10.1038/s41467-020-18171-8 (PMC7455731; doi:10.1038/s41467-020-18171-8)
Supplement: Supplementary file 12 — Reporting Summary [file 41467_2020_18171_MOESM12_ESM.pdf]

nature research

Corresponding author(s): Zheng-Jiang Zhu

Last updated by author(s): Jul 30, 2020

Reporting Summary

Nature Research wishes to improve the reproducibility of the work that we publish. This form provides structure for consistency and transparency in reporting. For further information on Nature Research policies, see our Editorial Policies and the Editorial Policy Checklist.

Statistics

For all statistical analyses, confirm that the following items are present in the figure legend, table legend, main text, or Methods section.

n/a	Confirmed	
☐	☒	The exact sample size (*n*) for each experimental group/condition, given as a discrete number and unit of measurement
☐	☒	A statement on whether measurements were taken from distinct samples or whether the same sample was measured repeatedly
☐	☒	The statistical test(s) used AND whether they are one- or two-sided<br>*Only common tests should be described solely by name; describe more complex techniques in the Methods section.*
☒	☐	A description of all covariates tested
☒	☐	A description of any assumptions or corrections, such as tests of normality and adjustment for multiple comparisons
☐	☒	A full description of the statistical parameters including central tendency (e.g. means) or other basic estimates (e.g. regression coefficient) AND variation (e.g. standard deviation) or associated estimates of uncertainty (e.g. confidence intervals)
☒	☐	For null hypothesis testing, the test statistic (e.g. *F*, *t*, *r*) with confidence intervals, effect sizes, degrees of freedom and *P* value noted<br>*Give P values as exact values whenever suitable.*
☒	☐	For Bayesian analysis, information on the choice of priors and Markov chain Monte Carlo settings
☒	☐	For hierarchical and complex designs, identification of the appropriate level for tests and full reporting of outcomes
☒	☐	Estimates of effect sizes (e.g. Cohen's *d*, Pearson's *r*), indicating how they were calculated

Our web collection on statistics for biologists contains articles on many of the points above.

Software and code

Policy information about availability of computer code

Data collection	These tools were used in data collection: Agilent MassHunter Workstation Data Acquisition (Version B.08.00); ChemAxon MarvinSketch (Version 16.10.24); ALOGPS (Version 2.1); MS-FINDER(version 3.24); MetFrag CL (version 2.4.5-CL); CFM-ID (version 2.4); BioTransformer (version 1.0.8); OpenBabel (version 2.4.1); R package rcdk (V3.4.7.1); ClassyFire (http://classyfire.wishartlab.com/); MetCCS (http://www.metabolomics-shanghai.org/MetCCS/); DeepCCS (https://github.com/plpla/DeepCCS); Chemical translation service (http://cts.fiehnlab.ucdavis.edu/)
Data analysis	Agilent IM-MS Reprocessor (Version B.08.00); PNNL PreProcessor (Version 2018.06.02); Agilent Mass Profiler (Version 10.0);

For manuscripts utilizing custom algorithms or software that are central to the research but not yet described in published literature, software must be made available to editors and reviewers. We strongly encourage code deposition in a community repository (e.g. GitHub). See the Nature Research guidelines for submitting code & software for further information.

Data

Policy information about availability of data

All manuscripts must include a data availability statement. This statement should provide the following information, where applicable:

- Accession codes, unique identifiers, or web links for publicly available datasets
- A list of figures that have associated raw data
- A description of any restrictions on data availability

All raw data files can be accessed at MetaboLights (MTBLS1622 and MTBLS1693). The annotation results for all metabolomics datasets were provided in the Supplementary Data 7-8. These public databases were used in this study: CCS compendium (downloaded at March 6th, 2020); ISiCLE database (downloaded at Feb. 18th, 2020); KEGG (downloaded at Aug. 2nd, 2018); HMDB (downloaded at June 9th, 2018); LMSD (downloaded at July 11th, 2019); MINE (downloaded at Feb. 7th, 2018); DrugBank (downloaded at April 12th, 2019); DSSTox (downloaded at May 6th, 2019); UNPD (downloaded at June 13th, 2019). All compounds and their CCS

values can be accessed in AllCCS webserver (http://allccs.zhulab.cn/) with free registration. Source data (Figure 2e-j, Figure 3b-f, Figure 4a, Figure 4e-g, Figure 5d, Figure 6a-e) are provided with this paper.

Field-specific reporting

Please select the one below that is the best fit for your research. If you are not sure, read the appropriate sections before making your selection.

☒ Life sciences ☐ Behavioural & social sciences ☐ Ecological, evolutionary & environmental sciences

For a reference copy of the document with all sections, see nature.com/documents/nr-reporting-summary-flat.pdf

Life sciences study design

All studies must disclose on these points even when the disclosure is negative.

Sample size	The experiments of aging mouse described were performed with 10 independent samples for each group. No sample-size calculation was performed here, and 10 independent biological replicates represent enough biological variations in most biological studies.
Data exclusions	No samples were excluded from analysis in this study.
Replication	The aging mouse liver tissue samples were only collected and measured once. The standard biological samples (i.e. MEF cell, human plasma, fruit fly) were repeated 3-6 technical replications (injections). All attempts at replications were successful.
Randomization	For aging mouse liver datasets, animals were assigned randomly to young (36 weeks) and old groups (104 weeks). For other datasets, the randomization is not required, because only one standard sample was used.
Blinding	This study is not be blinded in data measuring and analyzing. The main aim of this study is to validate technology (qualitatively annotating known and unknown metabolites) instead of generating new biological conclusions. Therefore, we think that our study is acceptable in scientific community.

Reporting for specific materials, systems and methods

We require information from authors about some types of materials, experimental systems and methods used in many studies. Here, indicate whether each material, system or method listed is relevant to your study. If you are not sure if a list item applies to your research, read the appropriate section before selecting a response.

Materials & experimental systems

n/a	Involved in the study	
☒	☐	Antibodies
☐	☒	Eukaryotic cell lines
☒	☐	Palaeontology and archaeology
☐	☒	Animals and other organisms
☒	☐	Human research participants
☒	☐	Clinical data
☒	☐	Dual use research of concern

Methods

n/a	Involved in the study	
☒	☐	ChIP-seq
☒	☐	Flow cytometry
☒	☐	MRI-based neuroimaging

Eukaryotic cell lines

Policy information about cell lines

Cell line source(s)	RIPK1-/- MEFs cell lines (generated from RIPK1 KO mice) were provided from Prof. Junying Yuan's Lab (Chinese Academy of Sciences, Shanghai).
Authentication	The cell lines used were authenticated by genotyping and western blotting.
Mycoplasma contamination	The cell line is negative for mycoplasma contamination by Prof. Junying Yuan's Lab.
Commonly misidentified lines (See ICLAC register)	No commonly misidentified cells were used.

Animals and other organisms

Policy information about studies involving animals; ARRIVE guidelines recommended for reporting animal research

Laboratory animals	Mouse (strain, c57BL/6J; male; age, 36 weeks and 104 weeks); Wild-type fruit flies (strain, 5905; FlyBase ID, FBst0005905; male; age, 3 days and 30 days)

Wild animals	The study did not involve any wild animals.
Field-collected samples	The study did not involve samples collected from the filed.
Ethics oversight	The mouse tissue studies were approved by Animal Ethics and Welfare Management Committee of Interdisciplinary Research Center on Biology and Chemistry, Chinese Academy of Sciences (Shanghai, China).

Note that full information on the approval of the study protocol must also be provided in the manuscript.
